# Decimeter-Level Accuracy for Smartphone Real-Time Kinematic Positioning Implementing a Robust Kalman Filter Approach and Inertial Navigation System Infusion in Complex Urban Environments

**DOI:** 10.3390/s24185907

**Published:** 2024-09-11

**Authors:** Amir Hossein Pourmina, Mohamad Mahdi Alizadeh, Harald Schuh

**Affiliations:** 1 Faculty of Geodesy and Geomatics Engineering, K. N. Toosi University of Technology, Tehran 19697, Iran; pourmina@email.kntu.ac.ir; 2 German Research Center for Geosciences (GFZ), 14473 Potsdam, Germany; harald.schuh@gfz-potsdam.de; 3 Institute of Geodesy and Geoinformation Sciences, Technische Universität Berlin, 10587 Berlin, Germany

**Keywords:** smartphones, real-time kinematic positioning, inertial navigation system, RTK/INS fusion, urban complex environments, Samsung Galaxy S21 Ultra, Xiaomi Mi 8

## Abstract

New smartphones provide real-time access to GNSS pseudorange, Doppler, or carrier-phase measurement data at 1 Hz. Simultaneously, they can receive corrections broadcast by GNSS reference stations to perform real-time kinematic (RTK) positioning. This study aims at the real-time positioning capabilities of smartphones using raw GNSS measurements as a conventional method and proposes an improvement to the positioning through the integration of Inertial Navigation System (INS) measurements. A U-Blox GNSS receiver, model ZED-F9R, was used as a benchmark for comparison. We propose an enhanced ambiguity resolution algorithm that integrates the traditional LAMBDA method with an adaptive thresholding mechanism based on real-time quality metrics. The RTK/INS fusion method integrates RTK and INS measurements using an extended Kalman filter (EKF), where the state vector x includes the position, velocity, orientation, and their respective biases. The innovation here is the inclusion of a real-time weighting scheme that adjusts the contribution of the RTK and INS measurements based on their current estimated accuracy. Also, we use the tightly coupled (TC) RTK/INS fusion framework. By leveraging INS data, the system can maintain accurate positioning even when the GNSS data are unreliable, allowing for the detection and exclusion of abnormal GNSS measurements. However, in complex urban areas such as Qazvin City in Iran, the fusion method achieved positioning accuracies of approximately 0.380 m and 0.415 m for the Xiaomi Mi 8 and Samsung Galaxy S21 Ultra smartphones, respectively. The subsequent detailed analysis across different urban streets emphasized the significance of choosing the right positioning method based on the environmental conditions. In most cases, RTK positioning outperformed Single-Point Positioning (SPP), offering decimeter-level precision, while the fusion method bridged the gap between the two, showcasing improved stability accuracy. The comparative performance between the Samsung Galaxy S21 Ultra and Xiaomi Mi 8 revealed minor differences, likely attributed to variations in the hardware design and software algorithms. The fusion method emerged as a valuable alternative when the RTK signals were unavailable or impractical. This demonstrates the potential of integrating RTK and INS measurements for enhanced real-time smartphone positioning, particularly in challenging urban environments.

## 1. Introduction

Reports indicate the substantial growth in location-based services (LBSs), which rely on precise positioning. This expansion has led to services catering to professionals and the general public. Among the various technologies available, Global Navigation Satellite Systems (GNSSs) have prominently emerged as the favored choice for ensuring accuracy within LBSs due to their remarkable capacity to function seamlessly across diverse weather conditions. Furthermore, GNSSs can seamlessly scale on a massive level while maintaining precision [1,2]. As reported within the 2022 GNSS market report by the European Union Agency for the Space Programme (EUSPA), most—over 92%—of the low-cost GNSS chips are used in smartphones. This prevalence is attributed to the widespread integration and use of smartphones, owing to their portability, affordability, and ease of access. This establishes them as the most prevalent LBS services [3]. A significant advancement in this realm was materialized by the introduction the GNSS raw observation API in 2016, aligning with the need for smartphone positioning. This innovation satisfied enhanced performance demands by providing essential metrics such as the pseudorange, carrier phases, Doppler effects, and signal-to-noise ratios (SNRs). As a result, numerous smartphone users have expressed keen interest in adopting more precise positioning techniques [4].

During the initial stages of GNSS development, a limited number of smartphones and tablets could conduct measurements, typically utilizing only a single frequency and one GNSS constellation. Notable among these devices were the Google Nexus 9, Samsung S8, and Huawei P9. However, a crucial moment arrived in 2018, when the Xiaomi Mi8 introduced a dual-frequency GNSS smartphone, catalyzing a transformative shift within the market landscape. This marked the inception of a new era where flagship smartphones embraced GNSS chipsets equipped with multiple frequencies and constellations, setting a new industry standard. Contemporary research endeavors focus on advancing positioning algorithms, particularly precise point positioning (PPP) and real-time kinematic (RTK) techniques. These advancements aim to achieve new levels of accuracy in positioning with smartphones. Despite these advancements, challenges still exist due to the inherent limitations posed by smartphone hardware. The utilization of tiny non-directional linearly polarized antennae and low-power receiver chipsets in smartphones has notably reduced the quality of GNSS signals. A number of factors contribute to the intricate challenge of achieving precise positioning while using smartphones, such as low signal-to-noise ratios (SNRs), increased pseudorange noises, and poor phase continuity [5].

The impact of linearly polarized antennae on raw observations became prominently evident when the GNSS antenna signal of a smartphone was linked to an external software receiver [6]. In-depth investigations conducted by [7,8], leveraging specially designed GNSS smartphone chipsets, showed the following concerning trend: raw observations exhibited an unusually elevated gross error ratio and remained susceptible to the detrimental influence of multipath effects. As a result of this outcome, the differential positioning accuracy achieved was confined to less than ten meters. Further exploration by [9] demonstrated the potential of time-differenced measurements, providing their capability to attain centimeter-level accuracy. This finding was particularly apparent after the duty cycle was brought to closure in the context of the Huawei P10 smartphone. Extensive evaluations, as carried out by [10,11,12], featured a detailed comparison of carrier-to-noise ratios (C/N0) and pseudorange noise against their geodetic receiver counterparts in the Nexus 9 tablet and Samsung S8 smartphone. These evaluations showed a substantial variation in the smartphone measurements. Refs. [13,14] confirmed this recurring theme by demonstrating that these smartphones perform poorly in GNSS signal reception. In studies conducted by [15,16], dual-frequency measurements were performed on the Xiaomi Mi8 smartphone, resulting in a significant breakthrough. Using L5/E5a signals, which possess extended wavelengths and high symbol rates, enabled measurements with enhanced accuracy and reduced sensitivity to multipath interference. Ref. [17] identified that the initial phase bias (IPB) calibration was insufficient in certain smartphones, including the Nexus 9, Galaxy S8, and Honor 8, resulting in markedly elevated levels of uncertainty. As a result, the importance of proper GNSS module calibration in mitigating accuracy discrepancies is evident.

A thorough investigation has been conducted concerning the comprehensive analysis of observations from multi-frequency and multi-constellation smartphones. The previous studies examined the GPS L1/L5, Galileo E1/E5a, BDS B1I, and GLONASS missions. The research outcomes revealed a notable consistency in the signal power, satellite visibility, pseudorange, and carrier-phase noise. However, there remains a research void regarding the quality attributes of the data and the positioning capabilities associated with the novel signals from the BDS-3. The unavailability of raw observation data from BDS-3 in smartphones has led to a research gap in understanding these specific signals. In addressing the challenges related to multipath interference within smartphone GNSS data, diverse algorithms have been evaluated. These encompass algorithms like the CMCD (Code-Minus-Carrier Delta range) and SNR elevation-dependent methods [18]. Remarkably, implementing these techniques removed over 77% of the observed multipath effects, yielding a substantial enhancement in the accuracy of the positioning outcomes.

The BCM47755 chipset, a groundbreaking development by Broadcom, marked a significant milestone in GNSSs. It was unveiled as Broadcom’s inaugural dual-frequency GNSS chipset in San Jose, California. This chipset heralded a new era of enhanced communication between smartphones and conventional GNSS receivers, offering advantages to users and developers alike. Introduced in May 2018, the BCM47755 chipset made its debut through Xiaomi, a prominent company in the smartphone industry. This strategic collaboration introduced a cutting-edge component that revolutionized location-based services on smartphones. By leveraging the capabilities of the dual-frequency GNSS chipset, Xiaomi smartphones could establish more robust and accurate connections with satellite-based positioning systems. Specifically, the BCM47755 chipset was designed to work seamlessly with L1/E1 and L5/E5 frequencies. This dual-frequency feature improved the accuracy of the location data and mitigated the impact of signal disruptions and inaccuracies caused by atmospheric conditions and signal reflections. In subsequent developments, researchers advancing GNSS receiver techniques for Android smartphones made significant progress. Among these advancements, Fortunato [15] and his team explored and implemented techniques such as RTK positioning and PPP on the Xiaomi Mi8 smartphone. 

Using RTK and PPP techniques, the dynamic positioning of the Xiaomi Mi8 was achieved, with impressive results. The horizontal root mean squares (RMSs), a key metric for assessing positioning accuracy, were reported as 1.17 m and 2.23 m for the RTK and PPP techniques, respectively. This demonstrated the chipset’s ability to deliver highly accurate and reliable positioning information, significantly outperforming conventional single-frequency GNSS solutions. The successful application of advanced GNSS receiver techniques showcased the capabilities of the BCM47755 chipset and demonstrated the potential for significantly enhancing smartphone-based positioning methods. With improved accuracy and reduced errors, these advancements hold great potential for many applications, from navigation and positioning to augmented reality and location-based services.

Previously, studies [5,6,8,19,20] used pseudorange differential corrections in the position domain using a measurement matrix, enabling meter-level positioning regardless of raw smartphone data. As a result of subsequent studies, greater precision has been achieved regarding positioning. As a result of leveraging dual-frequency raw observations derived from smartphones, a PPP model without the ionosphere was presented in [5]. The model utilized dual-frequency raw observations from smartphones to achieve sub-meter-level positioning under static open-sky conditions following convergence. Multipath effects have been demonstrated in a number of studies [5,21]. In their study [6], Pesyna et al. showed that it was possible to mitigate the multipath susceptibility of the built-in antennae of smartphones and achieve accurate positioning to the centimeter level by using an attached external auxiliary antenna. According to a recent research study [20,21], using smartphone antennae and a Kalman filter to link the temporal parameters (the multi-epoch model) to the internal antennae has achieved centimeter-level positioning accuracy. Carrier-phase drift in smartphone observation data has recently been confirmed by [22,23]. The phase offsets and drifts observed in this study are similar to those observed in previous studies (e.g., [5,6,8,13,24,25]).

It has been demonstrated that MEMS (Micro-Electro Mechanical System)-IMUs (Inertial Measurement Units) are affordable and capable of achieving accuracy levels within the decimeter range in 60 s when combined with high-precision GNSS receivers operating in the PPP mode during interruptions of the GNSS signals [26]. Combined with low-cost, single-frequency GNSS receivers, these devices can provide accuracy at the meter level [27]. To achieve decimeter-to-meter accuracy during 30 s of signal loss with four visible satellites, newer, more affordable dual-frequency GNSS receivers are integrated with MEMS-IMUs. It is estimated that dual-frequency processing provides a tenfold improvement over single-frequency processing [28]. Due to the availability of GNSS chipsets for smartphones, native sensor fusion methods that utilize smartphones’ onboard inertial sensors have been investigated. Using PPP, RTK, and other accurate techniques, smartphone positioning with sub-meter-to-decimeter accuracy is achievable with static positioning in open-sky environments [29,30,31,32,33]. However, obtaining precise and continuous positioning with smartphone raw observations in complex urban environments remains challenging. This is due to the frequent pseudorange gross errors, carrier-phase discontinuities, and the limited availability of GNSS ambiguity-fixed results in actual kinematics. 

Recent advancements in GNSSs have significantly improved location-based services across various applications. However, the accuracy of GNSSs can be compromised in urban canyons, areas with dense foliage, or environments characterized by a high building density. In these scenarios, INSs provide crucial navigation data independently of GNSS signals, ensuring continued accuracy and reliability. Recognizing the complementary strengths of GNSSs and INSs, this study investigates the different methods of integrating INS observations with GNSS data. By comparing these methods, we aim to identify the most effective approach to enhance the navigation accuracy in challenging environments—a step forward in applications ranging from autonomous driving to urban planning. Consequently, this paper evaluates various data processing methods based on INS observations, assessing their performance in typical GNSS-compromised conditions and illustrating their broader implications for precise navigation solutions. The objective of this study is (1) to increase the accuracy of positioning moving vehicles in complex urban environments using Android smartphones; (2) to process the collected data from two Android smartphones using SPP and RTK positioning methods, and fusion methods utilizing GNSS raw observations of varying frequencies and constellations and INS observations; (3) a comparison of the results of the processed smartphone data with the U-Blox GNSS receiver. 

## 2. Methodology

This section provides an introduction to the RTK measurement model, including the signal state check and observation consistency check. Afterward, the INS dynamic model is introduced utilizing smartphones’ INS sensors (the InvenSense ICM-20690 IMU model for the Xiaomi Mi 8 (Xiaomi, Beijing, China) and ST-Microelectronics LSM6DSO IMU model for the Galaxy S21 Ultra (Samsung, Suwon-si, Republic of Korea). Then, we use a robust Kalman filter to integrate the INS dynamic model and RTK measurement model.

### 2.1. Model of RTK Measurement

There are a number of differential GNSS (DGNSS) positioning methods available, but RTK positioning is one of the most popular. Operating in a situation with an open sky can achieve an accuracy of centimeters. In order to mitigate errors along the signal path, a base station’s aiding information can be utilized. Thus, it is possible to make double-difference measurements of both the pseudorange and carrier phase with this method. Our study has led to a standardization framework for the multi-constellation RTK positioning based on the undifferenced pseudorange and carrier-phase models, which can be summarized as follows:

(1) $$\left\{ \begin{array}{l} P=\rho +c\left(dt_{r}-dt^{s}\right)+Iono+Trop+\varepsilon_{P}\\ \lambda \Phi =\rho +c\left(dt_{r}-dt^{s}\right)+\lambda N-Iono+Trop+\varepsilon_{\Phi } \end{array}\right.$$

where *P* and *Φ* represent the pseudorange and carrier-phase measurements, respectively. The variable *ρ* is the geometric distance between the satellite and ground receiver, and the variables *dt^s^* and *dt_r_* are the offsets of the satellite and receiver clocks, respectively; *λ* and *N* relate to the wavelengths and ambiguities in carrier-phase measurements, respectively. The *Iono* and *Trop* terms also indicate the delays caused by the ionosphere and troposphere, respectively. The terms *ε_P_* and *ε_Φ_* encompass the measurement noise and other unmodeled errors.

The double-differenced measurement model can be obtained using the following method between the base station (*b*) and the rover station (*r*):

(2) $$\left\{ \begin{array}{l} \nabla \Delta P_{r,b}^{S_{i},S_{j}}=\nabla \Delta \rho_{r,b}^{S_{i},S_{j}}+\nabla \Delta_{\varepsilon r,b}^{\;S_{i},S_{j}}\\ \lambda \nabla \Delta \Phi_{r,b}^{S_{i},S_{j}}=\nabla \Delta \rho_{r,b}^{S_{i},S_{j}}+\lambda \nabla \Delta \Phi_{r,b}^{S_{i},S_{j}}+\nabla \Delta_{\varepsilon \Phi r,b}^{\;S_{i},S_{j}} \end{array}\right.$$

where $\{\nabla \Delta {\left(.\right)}_{r,b}^{S_{i},S_{j}}=\left({\left(.\right)}_{r}^{S_{i}}-{\left(.\right)}_{b}^{S_{i}}\right)-\left({\left(.\right)}_{r}^{S_{j}}-{\left(.\right)}_{b}^{S_{j}}\right)$ denotes the double-differenced operator.

It is possible to effectively eliminate clock offsets, *dt^s^*, and *dt_r_*, by using the double-differenced operations described in Equation (2). It is also possible to ignore the remaining double-differenced ionospheric and tropospheric delays, *Iono* and *Trop*, if the short baseline is less than 5 km [34]. This simplification assumes that, over short distances, the atmospheric conditions experienced by both the base station and the rover are similar enough that their differential effect can be neglected. This is a common assumption in RTK positioning to reduce the complexity and focus on the primary sources of error that can be more significant over short distances.

GNSS receivers come equipped with phase-lock loops (PLLs), but these PLLs have the following limitation: they cannot directly establish values for the carrier-phase measurements, a parameter known as ambiguity *N*. Consequently, this leads to the uncertain measurements of the carrier phase, introducing ambiguity. For precise carrier-phase measurements, it is most important to accurately address and resolve this ambiguity [35]. Achieving a high level of accuracy in RTK positioning depends on effectively dealing with this ambiguity. A technique called least-squares ambiguity decorrelation adjustment (LAMBDA) [36] has been used to solve this problem. This method adopts an integer least-squares approach to estimate the ambiguities. This technique optimizes the subsequent objective function using the least-squares methodology, as shown below:

(3) $$f\left(\nabla \Delta N\right)=min\left\{{\left(\nabla \Delta \hat{N}-\nabla \Delta N\right)}^{T}P_{\nabla \Delta \hat{N}}\left(\nabla \Delta \hat{N}-\nabla \Delta N\right)\right\}$$

Here, $\nabla \Delta \hat{N}$ and $P_{\nabla \Delta \hat{N}}$ represent the real-valued estimates for the ambiguity and its corresponding covariance matrix, respectively. Once the optimal integer ambiguity, $\nabla \Delta \overset{\cup }{N}$, is determined, it will be employed to update the receiver position, ${\overset{⌢}{p}}_{r}$, in the following equation:

(4) $$\overset{\vee }{p}=\overset{\wedge }{p_{r}}-P_{\overset{\wedge }{p_{r}},\nabla \Delta \overset{\wedge }{N}}P_{\nabla \Delta \overset{\wedge }{N}}^{-1}\left(\nabla \Delta \overset{\wedge }{N}-\nabla \Delta \overset{\vee }{N}\right)$$

where $\overset{\wedge }{p_{r}}$ and $\overset{\wedge }{N}$ are regarded as the float solution, while $\overset{\vee }{p}$ and $\nabla \Delta \overset{\vee }{N}$ are the fixed solution. Moreover, the ratio test is used to ensure the accuracy of the fixed ambiguities by validating the probability of their correctness [36]. This is essential in RTK positioning, where resolving the integer ambiguities accurately is crucial for precise positioning. After determining the float solution ambiguity and its covariance matrix through the Least-squares AMBiguity Decorrelation Adjustment (LAMBDA) method, the optimal integer ambiguity is identified. The fixed ambiguity solution’s accuracy is then validated using the ratio test. The ratio test compares the squared residuals of the second-best-candidate solution to the best-candidate solution. If the ratio of these squared residuals is greater than a predefined threshold, the best candidate is accepted as the correct solution. This helps to ensure that the fixed ambiguities are indeed accurate, reducing the chances of incorrect ambiguity fixing, which can lead to significant positioning errors.

According to previous studies, the noise variation in the GNSS observations of a smartphone differs from that measured by geodetic quality equipment. It shows a weak correlation with the elevation but a strong correlation with the C/N0 [10]. To address this, we employed a pseudorange weighting model that considers the C/N0 and sets the weight ratio of the pseudorange to carrier phase as 2.78 × 10^−6^. This model can be represented as $\sigma_{i}=\sqrt{a+b.10^{-\left(C/N0\right)/10}}$, where *σ_i_* denotes the pseudorange noise, and parameters *a* and *b* must be determined for each piece of equipment at different frequencies and constellations. The calibration details for parameters *a* and *b* can be referred to in [11,12]. Of course, if the pseudorange precision is 1 m, setting the carrier phase precision to 2.78 × 10^−6^ m (or approximately 2.78 μm) is not realistic. This extremely small ratio is likely chosen to emphasize the relative reliability and precision of carrier-phase measurements over pseudorange measurements, not to imply an exact physical precision of the carrier phase.

It is essential to have reliable observations available in order to enhance smartphone RTK positioning performance. Especially in urban environments, pseudorange gross errors, as well as carrier-phase cycle slips, are common, resulting in a decrease in the accuracy and continuity of smartphones with RTK solutions. A data quality control strategy based on [37] is proposed as a solution that combines both prior detection and post-detection methodologies. We performed a data check based on the signal state in order to check the data against the results of our observation characteristic analysis, which was discussed earlier. The following steps are involved in this method:

(5) $$\left\{\begin{array}{c} \;C/N0\;<\;k_{1}\;\;\;\;\;\;\;\;\;\;\;\;\;\;\\ ELE.\;<\;k_{2}\;\;\\ ADRS==2\;or\;4 \end{array}\right.$$

In addition to these conditions, the observations deemed unreliable are those in which either the C/N0 is below the threshold (*k*_1_ = 20 dB-Hz) or the elevation is below the threshold (*k*_2_ = 15 degrees). A carrier phase is also considered unreliable if bit1 of the Accumulated Delta Range State (ADRS) equals 2 or 4. This metric indicates the status of the carrier phase measurements, which is critical for identifying the reliability of the data. Specifically, a carrier phase is considered unreliable if bit1 of the ADRS equals 2 or 4. This classification helps in data quality control by filtering out unreliable observations, thereby enhancing the accuracy and continuity of the RTK positioning solution. This mechanism ensures that only high-quality data, free from significant errors or slips, is used in the positioning calculations.

Following the data-checking method, we determined whether there was a correlation between the inter-epoch variation in the pseudorange and carrier phase and the Doppler measurements to assess the consistency of the observations. The following is a detailed description of the steps involved in the process:

(6) $$\left\{\begin{array}{l} \left|D_{t+1}-D_{t}\right|<k_{3}\\ \left|\left(\phi_{t+1}-\phi_{t}\right)+{\bar{D}}_{t+1,t}\cdot \Delta t\right|<k_{4}\\ |\left(P_{t+1}-P_{t}\right)+\lambda \cdot {\bar{D}}_{t+1,t}\cdot \Delta t|<k_{5}\\ {\bar{D}}_{t+1,t}=\frac{D_{t+1}+D_{t}}{2} \end{array}\right.$$

where *D* represents the Doppler measurements in this context. Equation (6) also denotes the average Doppler of the adjacent epochs. In the consistency check, we first assessed the reliability by examining the Doppler epoch difference. It is reliable if this difference is less than 100 cycles per second. Next, we compared the carrier-phase epoch difference with the average Doppler. If the difference between the two is less than (*k*_4_ = 2.5 *cycles*), it indicates the absence of a significant cycle slip. We also verified the pseudorange against the threshold (*k*_5_ = *λ*·*k*_3_). The threshold values used in Equation (6) were empirically determined based on extensive testing and data analysis to ensure reliable performance in various conditions.

### 2.2. Model of INS Measurement

For inertial navigation, the following three types of coordinate systems are commonly used: the inertial frame (I-frame), the Earth-centered Earth-fixed frame (E-frame), and the body frame (B-frame). In terms of the dead-reckoning algorithm, it can be expressed as follows:

(7) r˙INSeν˙INSeC˙be=vINSeCbefibb−2ΩieevINSe+geCbeΩebb

In Equation (7), the variables $r_{INS}^{e}$ and $\nu_{INS}^{e}$ stand for the position and velocity vectors of the INS within the E-frame reference frame. The value $C_{INS}^{e}$ corresponds to the direction cosine matrix, signifying the transformation from the B-frame to the Earth-fixed reference frame. The term $f_{ib}^{b}$ signifies the specific force vector experienced by the system in the Earth-fixed reference frame concerning the I-frame reference frame. Additionally, $\Omega_{ie}^{e}$ represents the skew-symmetric matrix associated with the Earth’s rotation vector $\omega_{ie}^{e}$ in relation to the inertial reference frame, but described within the Earth-fixed frame. The notation $g^{e}$ designates the gravity vector experienced in the Earth-fixed reference frame.

The symbol $\Omega_{eb}^{b}$ is indicative of the skew-symmetric matrix, while $\omega_{{eb}^{\prime }}^{b}$ and $\omega_{eb}^{b}$ can be expressed as follows:

(8) $$\omega_{eb}^{b}=\omega_{ei}^{b}+\omega_{ib}^{b}=\omega_{ib}^{b}-\omega_{ie}^{b}=\omega_{ib}^{b}-C_{e}^{b}\omega_{ie}^{e}$$

where $\omega_{ib}^{b}$ denotes the gyroscope angular velocity and $C_{e}^{b}$ denotes the inverse matrix of $C_{b}^{e}$.

By using the dynamic equation, the error state model of INS can be expressed as follows [38]:

(9) δr˙INSeδv˙INSeφ˙e=δvINSe−2ΩieeδvINSe+Cbefibb×φe−Cbeδfibb−Ωieeφe+Cbeδωibb

where $\left[(C_{b}^{e}f_{ib}^{b})\times \right]$ denotes the skew-symmetric matrix of $\left(C_{b}^{e}f_{ib}^{b}\right)$. The errors of the specific force and gyroscope angular velocity can be written as follows:

(10) $$f_{ib}^{b}=\delta b_{a}+\varepsilon_{a}$$

(11) $$\omega_{ib}^{b}=\delta b_{g}+\varepsilon_{g}$$

As a result, the linearized state equation of the INS can be written as follows:

(12) $$\left[\begin{array}{c} \delta {\dot{r}}_{INS}^{e}\\ \delta {\dot{v}}_{INS}^{e}\\ {\dot{\varphi }}^{e}\\ \delta {\dot{b}}_{a}\\ \delta {\dot{b}}_{g} \end{array}\right]\;=\left[\begin{array}{ccccc} 0 & I & 0 & 0 & 0\\ 0 & -2\Omega_{ie}^{e} & \left[(C_{b}^{e}f_{ib}^{b})\times \right] & -C_{b}^{e} & 0\\ 0 & 0 & -\Omega_{ie}^{e} & 0 & C_{b}^{e}\\ 0 & 0 & 0 & 0 & 0\\ 0 & 0 & 0 & 0 & 0 \end{array}\right]\left[\begin{array}{c} {\delta r}_{INS}^{e}\\ {\delta v}_{INS}^{e}\\ \varphi^{e}\\ \delta b_{a}\\ \delta b_{g} \end{array}\right]+\left[\begin{array}{cc} 0 & 0\\ {-C}_{b}^{e} & 0\\ 0 & C_{b}^{e}\\ 0 & 0\\ 0 & 0 \end{array}\right]\left[\begin{array}{c} \varepsilon_{a}\\ \varepsilon_{b} \end{array}\right]$$

where $\delta r_{INS}^{e},\delta \nu_{INS}^{e}$ and $\Phi^{e}$ denote the position, velocity, and attitude error vector in the E-frame; $\delta b_{a}$ denotes the bias errors of the accelerometer and gyroscope, respectively, and the processing noise vectors of the accelerometer and gyroscope biases, respectively.

### 2.3. Enhanced Ambiguity Resolution

We propose an enhanced ambiguity resolution algorithm that integrates the traditional LAMBDA method with an adaptive thresholding mechanism based on real-time quality metrics. This approach dynamically adjusts the ratio test threshold according to the current measurement quality, thereby improving the robustness of the ambiguity resolution under varying conditions.

The ratio test threshold *T* is adapted based on the C/N_0_ and the observed multipath effects. Equation (13) defines the adaptive threshold *T*, as follows:

(13) $$T=k_{0}+k_{1}\cdot \left(\frac{C/N0}{M}\right)$$

where *k*_0_ and *k*_1_ are empirical constants, *C/N*_0_ is the carrier-to-noise density ratio, and *M* is a measure of the multipath effect derived from the signal quality indicators.

### 2.4. INS and RTK Fusion

The fusion algorithm integrates RTK and INS measurements using a robust Kalman filter (EKF), where the state vector x includes the position, velocity, orientation, and their respective biases. The innovation here is the inclusion of a real-time weighting scheme that adjusts the contribution of the RTK and INS measurements based on their current estimated accuracy. To integrate the INS dynamic model with the RTK measurement model effectively, this study employs an EKF, which is specifically tailored to handle the non-linearities and uncertainties inherent in combining these two types of data. The robustness of the Kalman filter in this context is achieved through enhancements that include adaptive noise covariance to manage the varying quality of GNSS data in urban environments and the implementation of outlier rejection mechanisms to mitigate the impact of spurious measurements from both GNSS and INS systems.

The integration process begins with the establishment of a comprehensive state vector that includes position, velocity, and orientation components derived from both GNSS and INS systems. The state vector is updated using a prediction model based on the INS data and a correction step that incorporates GNSS measurements. The equations for the prediction and update steps are as follows:

$$\mathrm{Prediction}:\;{\hat{x}}_{k|k-1}=F_{k}{\hat{x}}_{k-1|k-1}+B_{k}u_{k}$$

$$\mathrm{Update}:\;{\hat{x}}_{k|k}={\hat{x}}_{k|k-1}+K_{k}\left(y_{k}-H_{k}{\hat{x}}_{k|k-1}\right)$$

where ${\hat{x}}_{k|k-1}$ is the a priori state estimate, *F_k_* is the state transition model, *B_k_* is the control input model applied to the control vector *u_k_*_,_ K_k_ is the Kalman Gain, *y_k_* represents the measurements from the GNSS, and *H_k_* is the measurement matrix. The system continuously recalibrates the noise matrices *Q* (process noise) and *R* (measurement noise) based on the real-time assessment of the data quality from both systems, enhancing the filter’s robustness and reliability. Through these mechanisms, the robust Kalman filter effectively synthesizes data from the GNSS and INS systems, providing a more accurate and reliable estimate of the vehicle’s position and orientation than could be achieved by either system alone.

In this study, the integration of RTK and INS measurements is enhanced by a real-time weighting scheme designed to dynamically adjust the contribution of each data source based on its current estimated accuracy. This approach is critical for maintaining high accuracy in environments where the reliability of GNSS signals fluctuates, such as urban canyons or densely built-up areas. The measurement accuracy for the RTK position is primarily assessed using metrics such as the SNR and the geometry of visible satellites, while the accuracy of INS data is evaluated based on the observed drift and bias over time. The real-time weighting scheme assigns a weight to each measurement based on the inverse of its variance, ensuring that more accurate measurements have a greater influence on the final position estimate. Mathematically, this can be expressed as follows:

(14) $$\omega_{RTK}=\frac{1}{\sigma_{RTK}^{2}}$$

(15) $$\omega_{INS}=\frac{1}{\sigma_{INS}^{2}}$$

where $\omega_{RTK}$ and $\omega_{INS}$ represent the standard deviations of the RTK and INS measurements, respectively.

The final fused position estimate is then computed as a weighted average of the RTK and INS estimates, as follows:

(16) $${\hat{x}}_{fused}=\omega_{RTK}.{\hat{x}}_{RTK}+\omega_{INS}.{\hat{x}}_{INS}$$

This dynamic adjustment process allows the system to respond to real-time changes in measurement quality. For instance, in scenarios where RTK signals degrade due to multipath effects or poor satellite visibility, the system automatically increases the reliance on INS data to maintain positioning accuracy. Conversely, when the RTK data are robust, they contribute more significantly to the final estimate, leveraging its potential for high precision.

This adaptive fusion method ensures that the overall positioning solution remains stable and accurate, even in challenging environments, where GNSS alone would struggle to provide reliable results.

### 2.5. State Vector and System Model

In the integration of RTK and INS measurements using the EKF, the state vector is a crucial component that encapsulates all of the necessary variables representing the system’s current state. This state vector allows the system to continuously update and refine the positioning and navigation solutions by incorporating data from both the GNSS (RTK) and INS subsystems.

The state vector *x* in this study is designed to comprehensively represent the system’s state, including key variables that are essential for accurate positioning and navigation. The state vector is defined as follows:

(17) $$x=\left[\begin{array}{l} p\\ \nu \\ \theta \\ b_{GNSS}\\ b_{INS} \end{array}\right]$$

where *p* represents the position vector, which includes the *x*, *y*, and *z* coordinates of the system, *v* denotes the velocity vector, which includes the velocities along the *x*, *y*, and *z* axes, θ stands for the orientation, typically represented as Euler angles or quaternions, which define the system’s attitude, and *b_GNSS_* and *b_INS_* are the bias terms associated with the GNSS and INS measurements, respectively. These biases account for any systematic errors or drifts that may affect the accuracy of the measurements.

The state vector is critical in maintaining an accurate and continuous estimate of the vehicle’s position and orientation, even when one of the measurement systems (e.g., the GNSS) is unreliable or temporarily unavailable.

The system model predicts the evolution of the state vector over time based on the vehicle’s kinematics and the sensor inputs. This prediction is central to the Kalman Filter’s operation, allowing it to anticipate the system’s state and subsequently correct it using real-time measurements.

The system model can be expressed as follows:

(18) $$\dot{x}(t)=f\left(x\left(t\right),u\left(t\right),\omega \left(t\right)\right)$$

where *f*(.) represents the system’s dynamic function, which defines how the state evolves over time, *u*(*t*) is the control input, such as the acceleration or steering angle, influencing the system’s movement, and ω(t) is the process noise, accounting for uncertainties or disturbances in the system model.

For instance, in vehicular navigation, the position and velocity are updated based on the current speed and heading, while the orientation is adjusted according to the gyroscope readings from the INS. The bias terms *b_GNSS_* and *b_INS_* are modeled as random walk processes to capture their gradual drift.

The discrete-time version of the system model used in the EKF can be written as follows:

(19) $$x_{k+1}=F_{k}x_{k}+B_{k}u_{k}+\omega_{k}$$

where *F_k_* is the state transition matrix, which is derived from the continuous-time dynamic function *f*(*⋅*), *B_k_* is the control input matrix, and $\omega_{k}$ represents the process noise at time step *k*.

The state vector and system model are integral to the Kalman filter framework, which uses these elements to predict the system’s state and then corrects it based on measurement updates. During the prediction step, the system model estimates the state vector’s evolution as follows:

(20) $${\hat{x}}_{k|k-1}=F_{k}{\hat{x}}_{k-1|k-1}+B_{k}u_{k}$$

Here, ${\hat{x}}_{k|k-1}$ is the predicted state estimate at time *k* given the state at time *k* − 1.

This predicted state is then corrected using the measurement update, where real-time GNSS and INS measurements are incorporated to refine the state estimate, as follows:

(21) $${\hat{x}}_{k|k}={\hat{x}}_{k|k-1}+K_{k}\left(y_{k}-H_{k}{\hat{x}}_{k|k-1}\right)$$

where *K_k_* is the Kalman Gain, determining the weighting of the measurement update, *y_k_* is the measurement vector at time *k*, and *H_k_* is the measurement matrix, linking the state vector to the actual measurements.

This integration ensures that the system remains accurate and reliable, even in challenging environments, by effectively combining the strengths of both the GNSS and INS systems.

### 2.6. Measurement Update

The EKF measurement update equation incorporates a dynamic weighting factor *ω*, which is calculated based on the current accuracy of RTK and INS measurements, as follows:

(22) $$K=PH^{T}{\left(HPH^{T}+R\right)}^{-1}$$

(23) x=x+K(z−Hx)

(24) P=(I−KH)P

In the above equations, *K* is the Kalman Gain, *H* is the measurement matrix, *R* is the measurement noise covariance matrix, *z* is the measurement vector, and *ω* is the dynamic weighting factor, calculated as follows:

(25) $$\omega =\frac{\sigma_{INS}}{\sigma_{RTK}+\sigma_{INS}}$$

where *σ_RTK_* and *σ_INS_* are the current standard deviations of the RTK and INS measurements, respectively.

**Figure 1:**
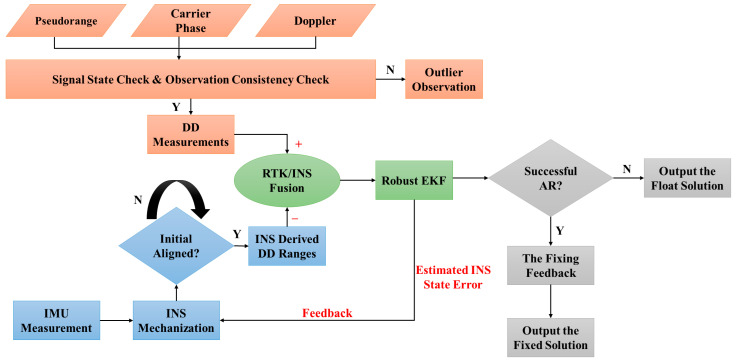
Flowchart depicting the TC RTK/INS integration architecture.

In addition to the details above, it is vital to highlight the essential elements encompassed within the tightly coupled (TC) RTK/INS fusion framework. This fusion approach involves critical components, including the mechanization and initial alignment of the INS system. To integrate the INS system effectively, it must convert its readings into comprehensive position, velocity, and attitude values. The position and velocity data from the navigation frame are then translated into INS measurements. Within the tightly coupled RTK/INS fusion process, the EKF calculations are based on the outcomes of the INS mechanization. Notably, this fusion scheme operates in a feedback loop, employing the state error estimations derived from the INS mechanization to counteract long-term drift. This mechanism is executed at the same frequency as the prediction step, where updates are performed less frequently than predictions. The rotation matrix for both frames is determined during the initial alignment phase, which encompasses the establishment of the body and navigation frame attitudes. After completing this alignment within the TC RTK/INS fusion, it is recommended to activate the fusion process. The TC RTK/INS fusion can yield three distinct outcomes based on its outputs. When valid GNSS measurements are inaccessible, the INS mechanization outcomes will be directly utilized. In such cases, the EKF estimates the errors associated with the states and feeds these errors back into the INS mechanization process, ultimately generating a float solution. Alternatively, a fixed INS solution is obtained by resolving carrier-phase ambiguities using the LAMBDA method, which identifies the optimal integer ambiguity set. This process involves initial float solution ambiguity estimation followed by validation through a ratio test to ensure the correctness of the fixed ambiguities. The detailed process of the ambiguity resolution is implemented within the smartphone’s RTK positioning algorithm. The entire methodology flowchart is visually depicted in Figure 1.

## 3. Data Used

**Figure 2:**
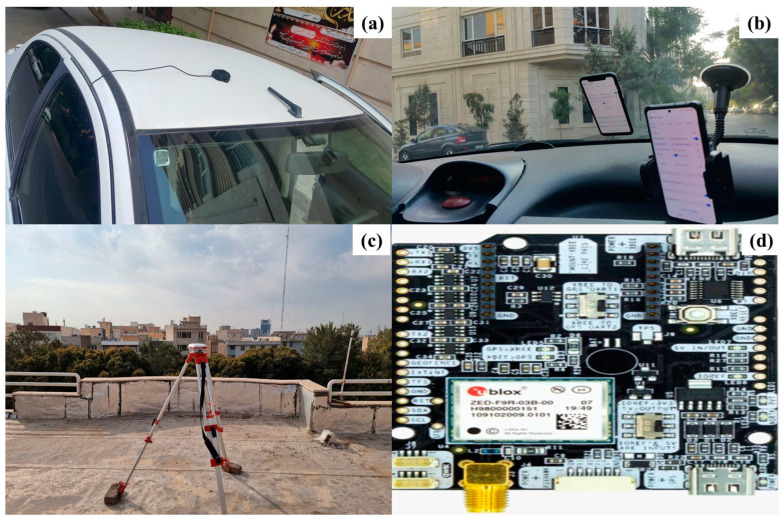
The U-Blox antenna installed on the car roof (**a**), the Xiaomi Mi8 (left) and Samsung Galaxy S21 Ultra (right) mounted in the car (**b**), the Stonex S3II SE geodetic receiver (**c**), and the ZED-F9R receiver chipset used for the U-Blox antenna (**d**).

**Figure 3:**
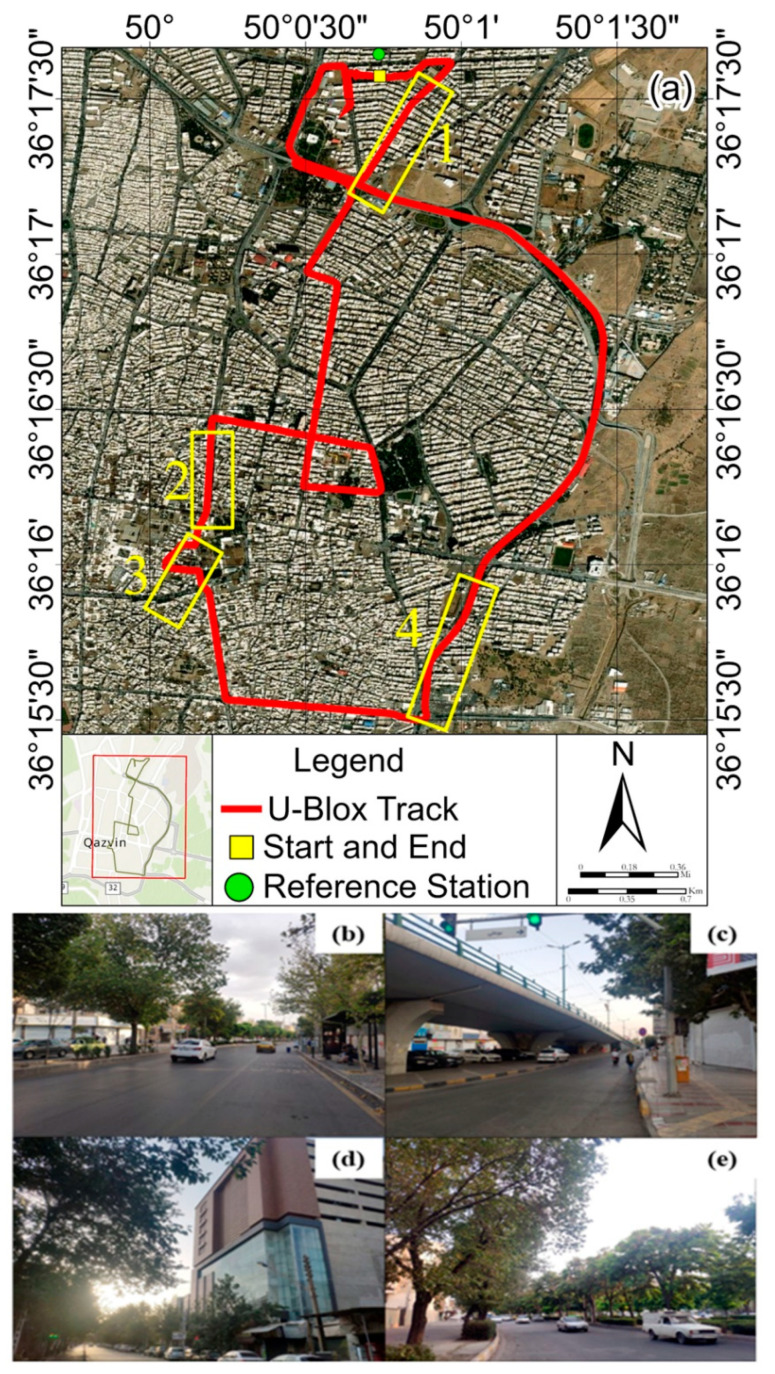
The vehicle’s path while logging measurements using smartphones and the U-Blox receiver (**a**). (**b**–**e**) Field photos of the 4 selected parts of paths 1, 2, 3, and 4 in (**a**), respectively.

With the Xiaomi Mi 8 and Samsung Galaxy S21 Ultra, dual-frequency signals (L1 and L5) can be tracked in addition to multiple constellations (GPS, GLONASS, Galileo, and BeiDou). An application called GnssLogger (Google) 3.0.6.4 version was used to simultaneously record the INS measurements and the raw GNSS observations for the experiment. A more realistic driving simulation can be achieved by mounting the smartphones on the dashboard rather than the vehicle’s roof (Figure 2b). This study assumes that the INS frame remains stationary with respect to the vehicle’s frame throughout. The Xiaomi Mi 8 is also equipped with the InvenSense ICM-20690 IMU model, while the Samsung Galaxy S21 Ultra uses the ST-Microelectronics LSM6DSO IMU model. This study was a Stonex geodetic receiver, model S3II SE, manufactured by Stonex, located in Milan, Italy as a reference station (Figure 2c). The car’s maximum distance from the reference station was 2 km. This is because, for short baselines of less than 5 km, the additional double-differenced ionospheric and tropospheric delays can also be disregarded [39,40]. On the other hand, the U-Blox receiver, model ZED-F9R, manufactured by U-Blox, located in Thalwil, Switzerland, which was placed on the car’s roof by an antenna (Figure 2a,d), and connected to Shamim’s Continuously Operating Reference Stations (CORSs) network on the Internet, which was used to compare the positions obtained from smartphones with the desired methods. This study was accomplished in Qazvin City in Iran, with the entire path shown in Figure 3.

This study operates under the assumption that the IMU frame remains fixed in relation to the vehicle frame for the entire duration of the experiment. Smartphones come with inertial sensors designed for consumer use, and their specifications are outlined in Table 1. Consequently, these smartphone IMUs are expected to bridge shorter periods of navigation outage in GNSS-denied scenarios. Specifically, for the IMU featured in the Galaxy S21 Ultra model, the factory datasheet and existing literature do not provide information on in-run bias. Therefore, future research could involve static IMU testing to calculate the Allan variance.

## 4. Field Test Results and Discussions

**Figure 4:**
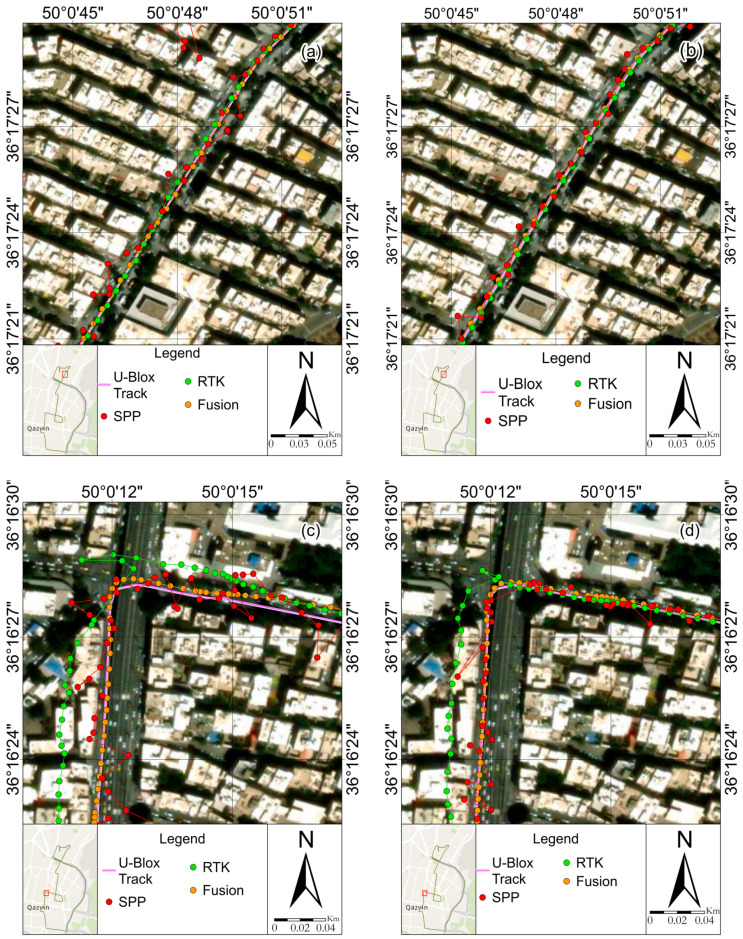

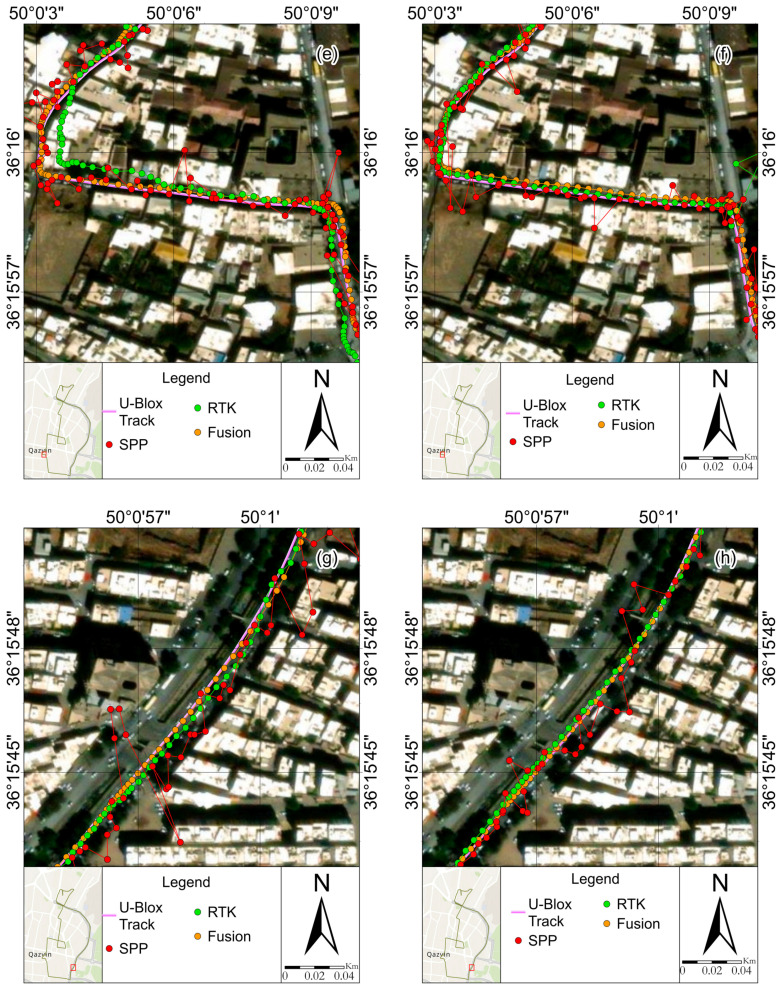
GNSS raw observations processed by three methods, SPP, RTK, and fusion, respectively, on four streets, Daneshgah, Naderi, Peighambarieh, and Khorramshahr. The figures on the right (i.e., **b**,**d**,**f**,**h**) show the results of the Mi 8, and the figures on the left (i.e., **a**,**c**,**e**,**g**) show the results of the S21 Ultra.

Field observations were processed using the RTKLIB for the SPP and RTK methods. For the RTK/INS fusion results, a separate custom software framework was used. This framework integrates GNSS data with INS measurements to enhance the positioning accuracy. We ensured that the quality control methods in our custom software aligned with those used in RTKLIB to make the results comparable. Specifically, both systems employ similar outlier detection and correction mechanisms. While the RTKLIB was used for the SPP and RTK processing, the RTK/INS fusion results were obtained using a custom software framework designed to integrate the GNSS and INS data effectively. We acknowledge that using different software for these processes could introduce variations due to differing quality control methods. However, we have aligned the quality control procedures between the RTKLIB and our custom software to ensure that the results are directly comparable. This approach allows us to leverage the strengths of each processing method while maintaining consistency in the data quality assessment. Next, the results are compared with the RTK/INS fusion method for two smartphones, the Xiaomi Mi 8 and Samsung S21 Ultra, using the U-Blox ZED-F9R receiver model. A sample case analysis was conducted on four segments of the overall vehicle trajectory. Figure 4 illustrates the trajectories of the results of three different methods, SPP, RTK, and RTK/INS fusion, respectively, on four streets, Daneshgah, Peighambarieh, Naderi, and Khorramshahr. The figures on the right (i.e., b, d, f, and h) show the results of the Mi 8, and the figures on the left (i.e., a, c, e, and g) show the results of the S21 Ultra. Also, Table 2 and Table 3 compare the horizontal error of the positioning results with the S21 Ultra and Xiaomi Mi 8 smartphones from the three SPP, RTK, and fusion methods with the U-Blox receiver.

**Figure 5:**
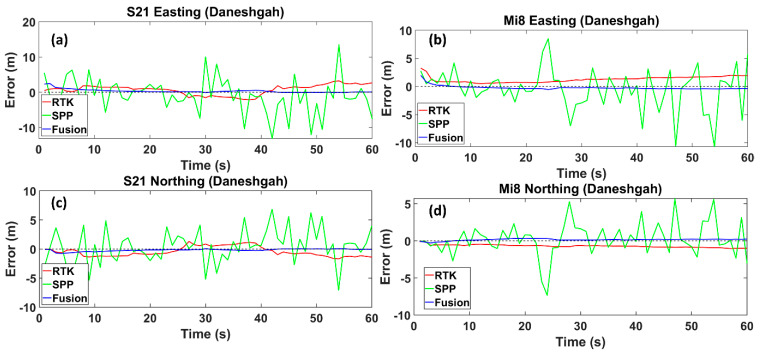
Easting and Northing errors regarding the reference trajectory for the Samsung Ultra S21 (**a**,**c**), and Xiaomi Mi8 (**b**,**d**), on Daneshgah Street, respectively. The dotted line is the zero-error axis.

The first selected street, Daneshgah, is a pretty wide street with low- and medium-sized buildings and an appropriate sight of the horizon. As expected, the street shows the lowest RMS values among other streets in both smartphones and for all methods. The RMS value in the fusion method compared to RTK mode was reduced by 74%, decreasing from 1.477 m to 0.380 m for the Xiaomi Mi8 smartphone. Likewise, there was a decline of 77% in the figure for the Samsung S21 Ultra, decreasing from 1.83 to 0.415. Compared to the SPP mode, the RMS from the fusion method value declined by 91% for the Xiaomi Mi8 smartphone, decreasing from 4.242 m to 0.380 m. In a similar trend, for the Samsung S21 Ultra, the RMS value for the SPP mode experienced a reduction of 92.8%, decreasing from 1.833 m to 0.415 m (Figure 5). It is worth noting that the RMS values of the RTK mode were smaller than those of the SPP mode for both smartphones. The SPP mode was less accurate than the RTK mode, which is expected, since SPP positioning typically uses signals from a single GNSS satellite constellation without corrections for atmospheric interference or other errors. The fusion method improved the SPP mode for both phones, which suggests that integrating data from additional sensors or systems (like IMUs or even other satellite constellations) can significantly enhance the positioning accuracy.

**Figure 6:**
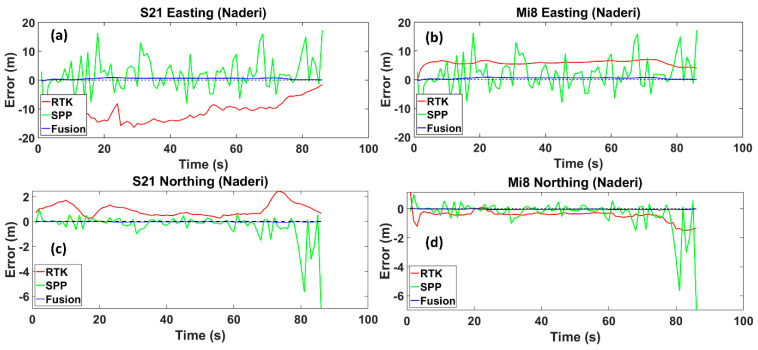
Easting and Northing errors regarding the reference trajectory for the Samsung Ultra S21 (**a**,**c**), and Xiaomi Mi8 (**b**,**d**), on Naderi Street, respectively. The dotted line is the zero-error axis.

The next street is Naderi. Compared to the previous street, it contains relatively higher buildings while also being wider. By passing under a bridge located at an intersection, the route enters the street (Figure 3c), which may overshadow the satellite geometry. As can be seen, the SPP and RTK modes, specifically for the Samsung S21 Ultra phones, show a lower accuracy than the fusion method on Naderi Street compared to the same modes on Daneshgah Street. This fact also applies to the fusion method on both streets to a lesser extent. It can be inferred that the complex urban environment highly affected the positioning of Naderi Street in the SPP and RTK modes. The fusion method reduces the RMS value by 90%, decreasing from 15.638 m to 1.518 m, compared to the RTK mode for the Xiaomi Mi8 smartphone. Similarly, there is a decrease of 85% in the figure for the Samsung S21 Ultra, declining from 16.356 m to 2.445 m. Compared to the SPP mode, the RMS value calculated from the fusion method reduces by 75% for the Xiaomi Mi8 smartphone, decreasing from 6.240 m to 1.518 m. Following a similar pattern for the Samsung S21 Ultra, the fusion method shows a decrease of 77% in the RMS value relative to the SPP mode. The RMS value from the fusion method is around 2.445 m, while those for the SPP mode are about 10.759 m (Figure 6). Both phones exhibit the smallest error margins with the RTK mode, suggesting high-precision capabilities when the RTK mode is available. The SPP mode shows the most significant error margins for both devices, which is typical without the RTK mode or correction data. The error is more influential on Naderi Street than Daneshgah Street, potentially due to environmental challenges such as more significant urban canyon effects or signal obstructions. The fusion method demonstrates an improvement over the SPP mode for both devices, with the error margins notably closer to the RTK mode. This method’s performance is consistent in both locations, suggesting robustness across different urban environments.

As expected, the visible satellites are reduced while passing under the bridge, and this affects the accuracy of the positioning in the RTK RMS values, as shown in Figure 4c,d. The reason why the fusion method overcomes this problem is the integration of RTK observations with INS observations, and, in some cases, the replacement of RTK observations with INS observations. Accordingly, the role of this integration becomes more prominent when the number of visible satellites is inadequate and INS observations are more reliable than RTK ones.

**Figure 7:**
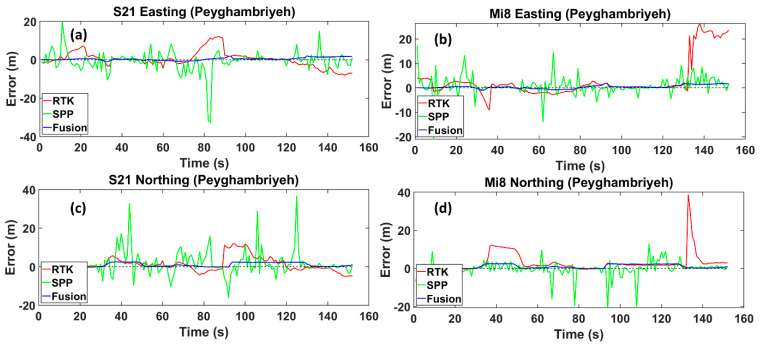
Easting and Northing errors regarding the reference trajectory for the Samsung Ultra S21 (**a**,**c**), and Xiaomi Mi8 (**b**,**d**), on Peyghambariyeh street, respectively. The dotted line is the zero-error axis.

Peighambarieh Street is a narrow street with some high-rise buildings. The RMS values in RTK mode for the Xiaomi Mi8 and Samsung S21 Ultra are around 9.805 m and 5.425 m, which, with the fusion method, decreased to 1.188 m and 1.515 m, respectively. These reductions of 88% and 72% are achieved by utilizing the fusion method instead of the RTK mode. Likewise, in the SPP mode, the results of the fusion method show upward trends of about 73% and 83% for the Xiaomi Mi8 and Samsung S21 Ultra, respectively. It is worth mentioning that the RMS values in the SPP mode are 4.484 m and 8.83 m, respectively (Figure 7). The results emphasize that the employed method is capable of overcoming various urban conditions. Both smartphones demonstrate precise positioning with the RTK mode, indicating that they can achieve high accuracy when RTK positioning is available. The SPP mode has the highest errors in both devices. This is expected, as the SPP mode typically does not use differential corrections and is thus more susceptible to errors due to atmospheric conditions, satellite geometry, and other factors. The fusion method consistently reduces the error compared to the SPP mode for both devices by effectively utilizing additional data sources or algorithms to enhance the accuracy.

**Figure 8:**
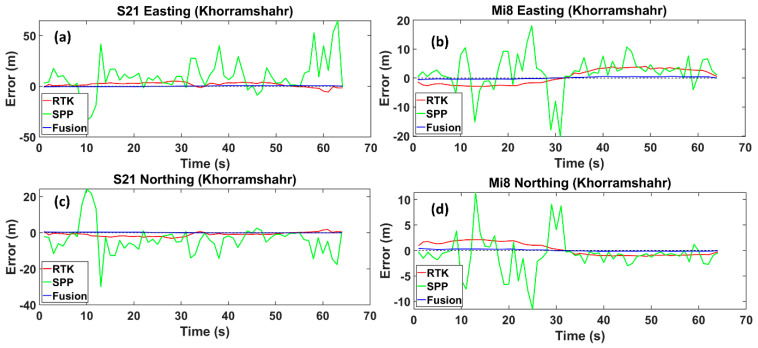
Easting and Northing errors regarding the reference trajectory for the Samsung Ultra S21 (**a**,**c**), and Xiaomi Mi8 (**b**,**d**), on Khorramshahr Street, respectively. The dotted line is the zero-error axis.

Finally, Khorramshahr Street is the widest street, with some high-rise buildings and occasional traffic congestion. The RMS value in the fusion method, compared to the RTK mode, was reduced by 84%, declining from 2.826 m to 0.446 m for the Xiaomi Mi8 smartphone. Similar to the Xiaomi Mi8 smartphone, there was a 76% decrease in the RMS values for the Samsung S21 Ultra, reducing from 2.955 m to 0.695 m. The RMS values for the SPP mode were 7.221 m and 21.382 m for the Xiaomi Mi8 and Samsung S21 Ultra, respectively. These values are higher than those of the fusion method by 94% and 97% (Figure 8).

**Figure 9:**
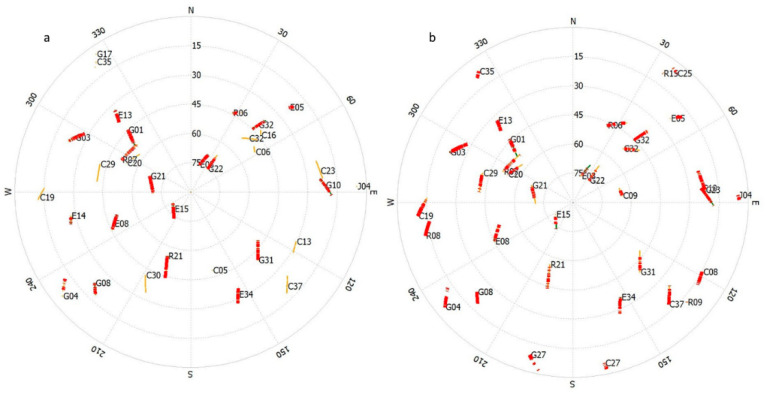
(**a**) Satellite sky plot from the Samsung S21 Ultra, and (**b**) satellite sky plot from the Xiaomi Mi8, including observations from the multi-GNSS in the first part of the operation.

Figure 9 represents the sky plots of the GNSS receiver for the Samsung Galaxy S21 Ultra smartphone and the Xiaomi Mi 8 smartphone, respectively. The colors indicate the quality of the signal received from each satellite, with warmer colors like red or yellow indicating a stronger signal and cooler colors like blue indicating a weaker signal. Based on these figures, one can assess the coverage and quality of the satellite signals being used for navigation. In complex urban environments, buildings and other structures can cause signal reflection and blockage, affecting both the number of visible satellites and the quality of the received signals, which, in turn, affects the accuracy of the position fix. The sky plot from the Xiaomi Mi 8 shows a good distribution of satellites; the actual navigation performance would depend on several factors, namely, satellite visibility, signal quality, and the GPS receiver’s capabilities. Without additional data, it is difficult to make a definitive statement on the comparative performance of the Xiaomi Mi 8 and the Samsung S21 Ultra solely based on the sky plots. The consistency of the data patterns across both devices indicates that environmental factors had a similar impact on both. The numerical data show that both smartphones can maintain a low level of error when using the RTK mode, typically within ±5 m and often within ±2 m. This suggests that both devices have high-quality GNSS antennae capable of leveraging RTK signals for precise positioning. The SPP errors are considerably larger, peaking at ±60 m for the S21 Ultra and ±20 m for the Mi 8. While this is partly due to the lack of RTK corrections, it also reflects the antennas’ ability to deal with urban challenges like multipath errors and signal obstructions. Dual-frequency GNSS receivers (such as the BCM47755 chipset) in the Xiaomi Mi8 and Samsung S21 Ultra help to mitigate noise and multipath errors. These receivers utilize signals from multiple frequencies (L1/E1 and L5/E5), which enhance the accuracy and reduce errors caused by atmospheric conditions and signal reflections. The IGG-III Weighting algorithm helps to detect and remove outliers by adjusting the weights based on the residuals, effectively mitigating the impact of noise and multipath errors in the data. Observations with larger residuals receive reduced weights or are discarded, which helps to maintain the accuracy. The fusion method reduces the error to ±10 m for both devices, suggesting that the antennae are able to provide sufficiently clean data that, when combined with other sensor inputs, result in much improved positional accuracy. The relatively small errors observed with the RTK mode suggest that both smartphones are equipped with antennae designed to minimize noise and maximize the capture of the direct signal from satellites, which is crucial for the RTK’s error correction algorithms to work effectively. Table 4 shows the results of the ambiguity resolution.

The ambiguity resolution process was tested using the Mi8 and S21 Ultra smartphones. The LAMBDA method and ratio test were employed to resolve carrier-phase ambiguities. The success rate indicates the percentage of epochs where ambiguities were correctly resolved, while the RMS error shows the residual error in meters after resolution. The results demonstrate high reliability and consistency across smartphones, validating the effectiveness of the proposed method.

## 5. Conclusions

Real-time positioning using raw GNSS measurements of smartphones is a new research topic in GNSS navigation. In this study, we used Samsung Galaxy S21 Ultra and Xiaomi Mi 8 smartphones to assess the conventional method’s accuracy and enhance the positioning accuracy using the INS fusion approach. Both smartphones can log code the pseudorange and carrier phase and Doppler measurements in two frequencies (L1/E1 and L5/E5) from the GPS, GLONASS, Galileo, BeiDou, and QZSS systems. Furthermore, a U-Blox GNSS receiver, model ZED-F9R, was used to compare the positions obtained from the smartphones using different approaches. Our method was based on the robust Kalman filter integration of the INS and raw GNSS measurements of the smartphones in the RTK mode. In urban areas with short or low-rise buildings, the RMS values were as low as the sub-meter level, i.e., 0.380 m for the Xiaomi Mi 8 smartphone and 0.415 m for the Samsung Galaxy S21 Ultra. However, in the worst case with complex urban environments and high-rise buildings, the RMS values for the Xiaomi Mi 8 and the Samsung Galaxy S21 Ultra smartphones increased to 1.518 m and 2.445 m, respectively.

Based on the results, both the Samsung Galaxy S21 Ultra and Xiaomi Mi 8 show the best performance with the RTK mode, which is known for its high accuracy in open-sky conditions and is often used in surveying and applications requiring high precision. The SPP mode has the largest errors, which is typical for urban environments where GNSS signals can be obstructed or reflected. The fusion method appears to be an effective compromise, significantly improving the SPP mode and approaching the accuracy of the RTK mode. It is important to note that these results are specific to streets with low-rise and open-sky buildings, and performance could vary in different environments. Also, on streets with high-rise buildings, the Samsung Galaxy S21 Ultra and Xiaomi Mi 8 showed the best performance with the RTK mode, which offers high precision but may not always be available in all urban environments. The SPP mode is less reliable with larger error margins, indicating the challenges posed by urban navigation. The fusion method consistently improves the SPP mode and provides a more stable error margin, which could be due to the integration of additional sensors or data sources. The overall trends suggest that both devices are capable of similar performance, with the specific method used for positioning having a significant impact on the accuracy. The SPP mode remains the least reliable, and the fusion method appears to be a robust alternative that offers improved accuracy over the SPP mode and which is close to the RTK performance. The consistent improvement seen with the fusion method in both locations suggests that it may be a viable option for urban navigation where the RTK mode is not available. On the other hand, on Khorramshahr Street, the positioning performance trends for both the Samsung Galaxy S21 Ultra and Xiaomi Mi 8 are similar to those observed on Daneshgah and Naderi Streets, with the RTK mode offering the highest precision. The SPP mode remains less reliable, while the fusion method consistently outperforms both the SPP and RTK methods, offering superior accuracy and robustness in challenging urban environments. These trends suggest that the fusion method could be a reliable approach for urban navigation when RTK signals are not optimally available.

From this research, it can be concluded that selecting the appropriate GNSS method is crucial, considering the environment and correct signal availability. Multi-sensor fusion techniques significantly enhance the positioning accuracy in complex urban areas. In most cases, the RTK mode outperformed the SPP mode, offering decimeter-level precision, while the fusion method bridged the gap between the two, showcasing improved stability accuracy. The fusion method, combining GNSS data with INS data, offers a robust alternative, approaching RTK accuracy and, in some cases, obtaining better accuracy. The Samsung Galaxy S21 Ultra and Xiaomi Mi 8 exhibited quality GNSS receivers, with minor performance differences, likely attributed to hardware and software variations. This study emphasizes the importance of choosing a suitable positioning method based on task-specific requirements. The impact of noise and multipath errors on the RTK accuracy is noted, with the fusion method mitigating these issues. The GNSS performance of smartphones depends on the antenna quality, hardware design, and software algorithms. Through advanced processing techniques, smartphones demonstrate high-precision GNSS capabilities in ideal conditions and reasonable performance in urban environments. Numeric performance metrics should be interpreted in the overall GNSS system performance context.

While this study has demonstrated the superior performance of the fusion method in complex urban environments, several areas warrant further investigation to enhance its application and robustness. Future research could explore the integration of additional sensor data, such as LiDAR or visual odometry, to further improve the accuracy and reliability of positioning in environments with severe GNSS signal degradation. Moreover, optimizing the real-time weighting scheme through machine learning techniques could dynamically adapt the fusion method to varying environmental conditions, potentially offering even greater precision. Investigating the application of the fusion method in different vehicle platforms, such as autonomous drones or marine vessels, could also expand its usability across diverse fields. Finally, a more detailed analysis of the impact of different types of urban infrastructure on the performance of the fusion method would provide valuable insights for tailoring the system to specific operational environments.

## Figures and Tables

**Table 1:** Factory specifications of the inertial sensors used.

Phone Models	Xiaomi Mi8	Samsung Galaxy S21 Ultra
IMU Model	InvenSense ICM-20690	ST-Microelectronics LSM6DSO
Gyroscope		
In-run bias stability (°/h)	>1000	-
Noise density (°/s/√Hz)	0.004	0.004
Standard full range (±°/s)	2000	2000
Accelerometer		
In-run bias stability (mg)	40	-
Noise density (μg/√Hz)	100	110
Standard full range (±g)	16	16

**Table 2:** Comparison of the horizontal error of the positioning results with the Mi 8 smartphone from the three SPP, RTK, and fusion methods with the U-Blox receiver.

St.	RTK (m)	SPP (m)	Fusion (m)
Daneshgah	1.477	4.242	0.380
Khorramshahr	2.826	7.221	0.446
Naderi	15.638	6.240	1.518
Peighambarieh	9.805	4.484	1.188

**Table 3:** Comparison of the horizontal error of the positioning results with the S21 Ultra smartphone from the three SPP, RTK, and fusion methods with the U-Blox receiver.

St.	RTK (m)	SPP (m)	Fusion (m)
Daneshgah	1.833	5.830	0.415
Khorramshahr	2.955	21.382	0.695
Naderi	16.356	10.759	2.445
Peighambarieh	5.425	8.830	1.515

**Table 4:** Ambiguity resolution results of the smartphones.

Measurement	Success Rate (%)	RMS Error (m)	Note
Mi8	95.2	0.03	High consistency achieved
S21 Ultra	96.0	0.02	Consistent with Doppler and TDCP

## Data Availability

The data presented in this study are available on request from the corresponding author.
